# Repurposing of nitroxoline as a potential anticancer agent against human prostate cancer – a crucial role on AMPK/mTOR signaling pathway and the interplay with Chk2 activation

**DOI:** 10.18632/oncotarget.5655

**Published:** 2015-10-03

**Authors:** Wei-Ling Chang, Lih-Ching Hsu, Wohn-Jenn Leu, Ching-Shih Chen, Jih-Hwa Guh

**Affiliations:** ^1^ The Division of Medicinal Chemistry, College of Pharmacy, Ohio State University, Columbus, OH 43210, USA; ^2^ School of Pharmacy, National Taiwan University, Taipei 100, Taiwan

**Keywords:** Nitroxoline, AMPK/mTOR signaling, Chk2, G1 arrest, Cyclin D1-Rb-Cdc25A axis

## Abstract

Nitroxoline is an antibiotic by chelating Zn^2+^ and Fe^2+^ from biofilm matrix. In this study, nitroxoline induced G1 arrest of cell cycle and subsequent apoptosis in prostate cancer cells through ion chelating-independent pathway. It decreased protein levels of cyclin D1, Cdc25A and phosphorylated Rb, but activated AMP-activated protein kinase (AMPK), a cellular energy sensor and signal transducer, leading to inhibition of downstream mTOR-p70S6K signaling. Knockdown of AMPKα significantly rescued nitroxoline-induced inhibition of cyclin D1-Rb-Cdc25A axis indicating AMPK-dependent mechanism. However, cytoprotective autophagy was simultaneously evoked by nitroxoline. Comet assay and Western blot analysis demonstrated DNA damaging effect and activation of Chk2 other than Chk1 to nitroxoline action. Instead of serving as a DNA repair transducer, nitroxoline-mediated Chk2 activation was identified to function as a pro-apoptotic inducer. In conclusion, the data suggest that nitroxoline induces anticancer activity through AMPK-dependent inhibition of mTOR-p70S6K signaling pathway and cyclin D1-Rb-Cdc25A axis, leading to G1 arrest of cell cycle and apoptosis. AMPK-dependent activation of Chk2, at least partly, contributes to apoptosis. The data suggest the potential role of nitroxoline for therapeutic development against prostate cancers.

## INTRODUCTION

Development of new chemical entities is costly and time-consuming. Drug repurposing (also known as drug repositioning), which refers to the discovery of new indications from existing drugs, has been a popular approach in recent years. After successful use of several non-anticancer drugs for cancer therapy, drug repurposing has attracted particular attention in both pre-clinical and clinical studies [1, 2].

Chk2 is a tumor susceptibility gene encoding for a serine/threonine protein kinase responsive to cellular DNA damage [3, 4]. Activated Chk2 protein kinase serves as a signal transducer, phosphorylating a wide variety of substrates including cell division cycle 25 (Cdc25) family members, promyelocytic leukemia protein (PML), E2F-1, BRCA1 (BReast CAncer gene 1) and p53, which have been highly associated with regulating DNA damage response and repair, cell cycle arrest as well as apoptosis [3–5]. During DNA damage response, Chk2 phosphorylates downstream substrates (e.g., BRCA1), leading to initiation of homologous recombination DNA repair signaling which may crossly interact non-homologous end joining repair pathway [6, 7]. When DNA damage cannot be repaired, damaged cells are able to introduce Chk2 kinase-mediated p53-dependent apoptotic programs [8]. Notably, Chk2 may also induce p53-independent apoptosis through phosphorylation at a Chk2 consensus phosphorylation site in E2F1, leading to protein stabilization, transcriptional activation and localization of phosphorylated E2F1 to discrete nuclear structures and induce apoptosis [9]. Based on the critical role during DNA damage response and cell apoptosis, Chk2 has been suggested as a potential target for anticancer therapy.

AMP-activated protein kinase (AMPK), existing as a heterotrimeric complex composed of a catalytic α subunit and regulatory β and γ subunits, acts as a metabolic master switch in regulating various intracellular processes including uptake of glucose, oxidation of fatty acids and biogenesis of mitochondria and glucose transporter 4 [10, 11]. Cellular stresses which result in depletion of cellular ATP and an increase in AMP:ATP ratio make AMPK a more susceptible substrate for phosphorylation in the activation loop of α subunit by the upstream AMPK kinase, LKB1. AMPK can also be phosphorylated and activated by calcium/calmodulin protein kinase kinase (CAMKK) 2 through changes in intracellular calcium levels [11–14]. Several lines of evidence reveal that AMPK and downstream mTOR pathways are potential therapeutic targets for the treatment of cancer [11, 13–15].

Nitroxoline, which functions by chelating Zn^2+^ and Fe^2+^ from biofilm matrix, is an antibiotic with effective activity at combating biofilm infections [16]. Nitroxoline has been suggested to induce intracellular oxidant stress [17]. Shim and the colleagues have performed a high throughput screening test for discovering the inhibitors of type 2 methionine amino peptidases (MetAP2) and have found that nitroxoline inhibited MetAP2 activity in human umbilical vein endothelial cells, leading to inhibition of endothelial tube formation and proliferation [18]. Furthermore, nitroxoline caused a reduction in tumor volume in breast cancer xenografts and in bladder cancer in an orthotopic mouse model [19]. Nitroxoline derivatives have been suggested to inhibit cathepsin B activity and to abrogate the invasion of Ras-transformed MCF-10A neoT cells [19]. However, the anticancer activity and mechanism of nitroxoline against human prostate cancer are yet to be identified. In this study, the role of AMPK/mTOR signaling pathway and the interplay with Chk2 in apoptosis have been identified in nitroxoline-mediated anticancer effect in both hormone-sensitive and hormone-refractory prostate cancer cells.

## RESULTS

### Nitroxoline induces anti-proliferative effect in human prostate cancer cells

**Figure 1:**
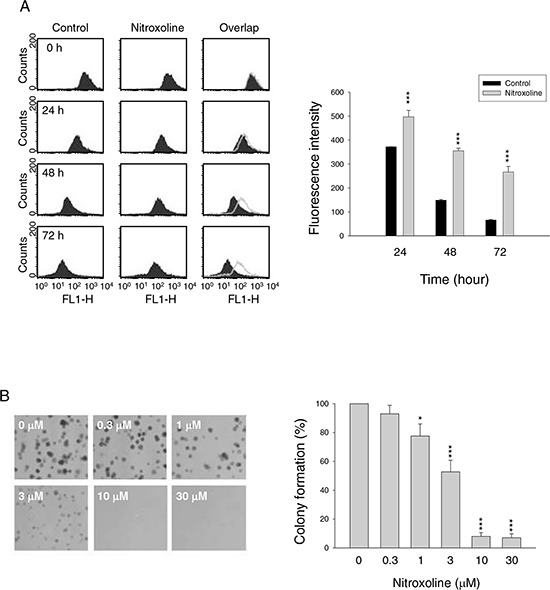
Effect of nitroxoline on anti-proliferation PC-3 cells were incubated in the absence or presence of nitroxoline (A, 10 μM; B, 0.3 to 30 μM) for the indicated time (A, 24 to 72 hours; B, ten days). After treatment, the cells were harvested for flow cytometric analysis of CFSE staining **A.** or the clonogenic assay was applied **B.** Data are representative of three independent experiments. Quantitative data are expressed as mean ± SEM of three independent experiments. *P < 0.05 and ***P < 0.001 compared with the control.

The anti-proliferative effects of nitroxoline in both hormone-sensitive (LNCaP) and hormone-refractory prostate cancer cells (PC-3 and DU-145) were determined using sulforhodamine B (SRB) assay. Nitroxoline induced time-dependent inhibition of cell proliferation in these cell lines (Table 1). Flow cytometric analysis of carboxyfluorescein succinimidyl ester (CFSE) staining was applied to further confirm anti-proliferative activity. The data demonstrated that nitroxoline prevented the decrease of fluorescence intensity in PC-3 cells, indicating the inhibition of cell proliferation (Figure 1A). Similar effect was obtained in both DU-145 and LNCaP cells (fluorescence intensity at 72 h of control *vs.* nitroxoline: 144 ± 4 *vs*. 326 ± 9, *P* < 0.001 in DU145; 258 ± 24 *vs*. 498 ± 15, *P* < 0.001 in LNCaP). The long-term effect of nitroxoline was examined using colonogenic assay. After a 10-day exposure, nitroxoline resulted in a profound inhibition of colony formation with an IC_50_ of 3.2 ± 0.6 μM (Figure 1B).

**Table 1:** Effect of nitroxoline on cell proliferation in human prostate cancer cells

Cell line	Time (hour)		
	24	48	72
PC-3	8.3±0.5	5.5±0.1	4.6±0.4
DU-145	16.6±1.0	7.4±0.3	5.5±0.1
LNCaP	6.6±0.3	5.0±0.3	4.2±0.1

Cells were incubated in the absence or presence of graded concentrations of nitroxoline for the indicated time. After the treatment, cells were fixed and stained for SRB assay. IC_50_ values (μM) are expressed as mean±SEM of three to four independent determinations.

### Inhibition of cyclin D1-Rb-Cdc25A axis contributes to nitroxoline-induced G1 arrest

**Figure 2:**
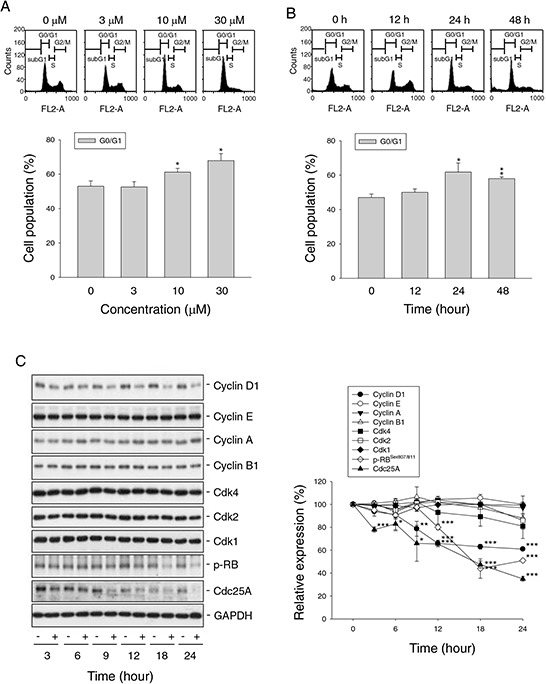
Effect of nitroxoline on cell cycle progression and related regulator proteins PC-3 cells were incubated in the absence or presence of nitroxoline (A, 3 to 30 μM; B, 10 μM; C, 10 μM) for the indicated time (A, 24 hours; B, 12 to 48 hours; C, 3 to 24 hours). The cells were fixed and stained with propidium iodide to analyze DNA content by flow cytometric analysis **A and B.** or the cells were harvested and lysed for the detection of the indicated protein by Western blot analysis **C.** Data are representative of three to four independent experiments. Quantitative data are expressed as mean ± SEM of three to four independent experiments. *P < 0.05, **P < 0.01 and ***P < 0.001 compared with 100% control.

Flow cytometric analysis of propidium iodide (PI) staining in PC-3 cells revealed that nitroxoline induced G1 arrest of the cell cycle (Figure 2A and 2B) followed by a significant increase of apoptosis after a 48-hour treatment (25.3 ± 0.6% compared with control of 4.7 ± 1.1%, *n* = 3, *P* < 0.001). Cell cycle progression is controlled by periodic activation of several Cdk/cyclin complexes. Cyclin D1 and its catalytic partner Cdk4 are key players in G1 phase progression. Nitroxoline induced a time-related decrease of protein expression of cyclin D1 other than cyclin E, A and B1 in PC-3 cells (Figure 2C). Rb protein, a tumor suppressor responsible for G1 checkpoint, blocks the entry of S phase and cell cycle progression. Cyclin D1/Cdk4 complex inhibits Rb by partial phosphorylation, decreasing its association with E2F transcription factor and allowing E2F-regulated activation of downstream gene transcription [20, 21]. As a result, nitroxoline decreased cyclin D1 protein expression, leading to the inhibition of Rb phosphorylation (Figure 2C). Furthermore, nitroxoline decreased Cdc25A protein expression (Figure 2C). Cdc25A, a member of Cdc25 family of dual-specificity phosphatases, is required for S phase entry and is induced in G1 phase by E2F [22, 23]. These data suggest that nitroxoline induces G1 arrest through the inhibition of cyclin D1-Rb-Cdc25A axis in PC-3 cells. Similar effects were obtained in LNCaP cells (Supplementary Figure 1).

### Nitroxoline regulates AMPK-mTOR pathway and induces autophagy

**Figure 3:**
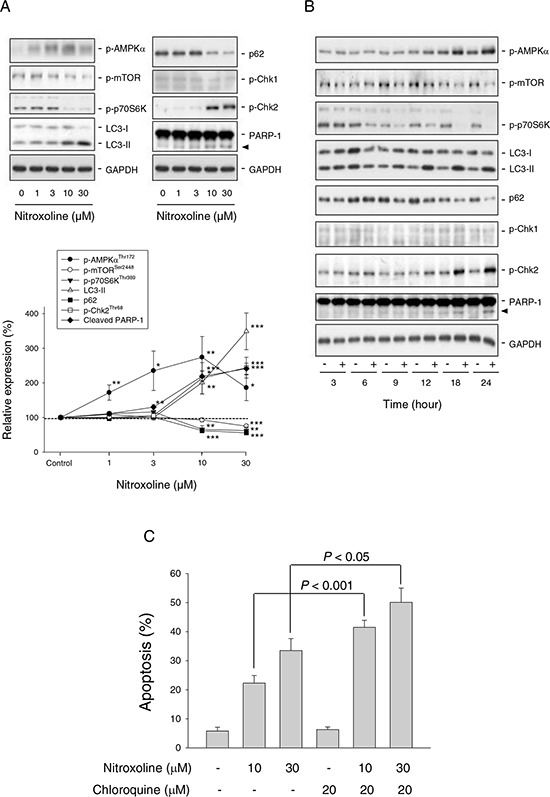
Effect of nitroxoline on the expression of several proteins and examination of autophagy-mediated cytoprotective effect PC-3 cells were incubated in the absence or presence of nitroxoline (A, 1 to 30 μM; B, 10 μM) for the indicated time (A, 24 hours; B, 3 to 24 hours). Cells were harvested and lysed for the detection of the indicated protein expression by Western blot analysis. Protein expression was quantified using computerized image analysis system ImageQuant (Amersham Biosciences, NJ, USA). Data are expressed as mean ± SEM of three to four independent experiments. *P < 0.05, **P < 0.01 and ***P < 0.001 compared with the control. **C.** PC-3 cells were incubated in the absence or presence of the indicated compound for 48 hours. The cells were fixed and stained with propidium iodide to analyze hypodiploid DNA content (apoptotic sub-G1 population) by flow cytometric analysis. Data are expressed as mean ± SEM of four independent experiments.

AMPK, a cellular energy sensor and signal transducer, is induced by a wide variety of metabolic stresses. mTOR, a downstream effector of AMPK, is a crucial player in cell growth and proliferation via regulating various cellular processes including transcription and translation [25]. Nitroxoline induced both concentration-dependent (Figure 3A) and time-dependent (Figure 3B) increases of AMPKα phosphorylation at Thr172 and decreases of mTOR phosphorylation at Ser-2448 in PC-3 cells. Nitroxoline also inhibited p70S6K phosphorylation at Thr389, a downstream substrate of mTOR (Figure 3A and 3B). The data indicate that nitroxoline induces the activation of AMPK but inhibition of mTOR signaling pathway in PC-3 cells. Similar effects were obtained in LNCaP cells (Supplementary Figure 1).

Autophagy is a catabolic process involving lysosomal degradation of dysfunctional or unnecessary cellular components. Autophagy is promoted by AMPK but inhibited by mTOR [26, 27]. Detection of LC3 is a reliable method for monitoring autophagy since lysosomal turnover of LC3-II reflects autophagic activity. Moreover, the level of p62 which is a ubiquitin- and LC3-binding protein is increased when autophagy is impaired [28]. As a result, nitroxoline induced a dramatic increase of LC3-II protein level but a decrease of p62 expression (Figure 3A and 3B), indicating an increase of autophagic activity. Increasing lines of evidence suggest that autophagy can serve as a survival pathway after cellular stresses by chemotherapy or radiation [27]. Chloroquine, an inhibitor of autophagy, was used to examine whether nitroxoline-induced autophagy represented a mechanism for cancer cells to survive or to promote programmed cell death. The data demonstrated that chloroquine, by itself, did not affect cell viability but significantly potentiated nitroxoline-induced cell apoptosis (Figure 3C and supplementary Figure 2), indicating a pro-survival role of autophagy.

**Figure 4:**
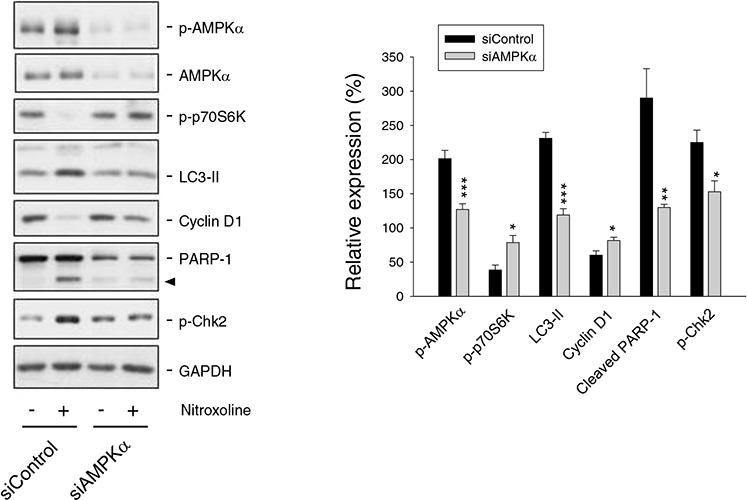
Examination of AMPK-regulated protein expression in PC-3 cells Both the control and AMPKα knockdown cells were treated with or without nitroxoline (10 μM) for 24 hours. Cells were harvested and lysed for the detection of the protein expression using Western blot analysis. The protein expression was quantified using computerized image analysis system ImageQuant (Amersham Biosciences, NJ, USA). The data are quantified relative to nitroxoline-free group and are expressed as mean ± SEM of three to four independent experiments. *P < 0.05, **P < 0.01 and ***P < 0.001 compared with the respective control.

siRNA-mediated knockdown of AMPKα was used to determine its functional regulation. AMPKα knockdown significantly inhibited nitroxoline-induced effects, including down-regulation of cyclin D1, inhibition of p70S6K phosphorylation, increase of LC3-II turnover and cleavage of PARP-1 (Figure 4). The data suggest that AMPK activation plays an upstream role to control cell cycle regulators and autophagic activity.

### Nitroxoline induces DNA damage and activates Chk2

**Figure 5:**
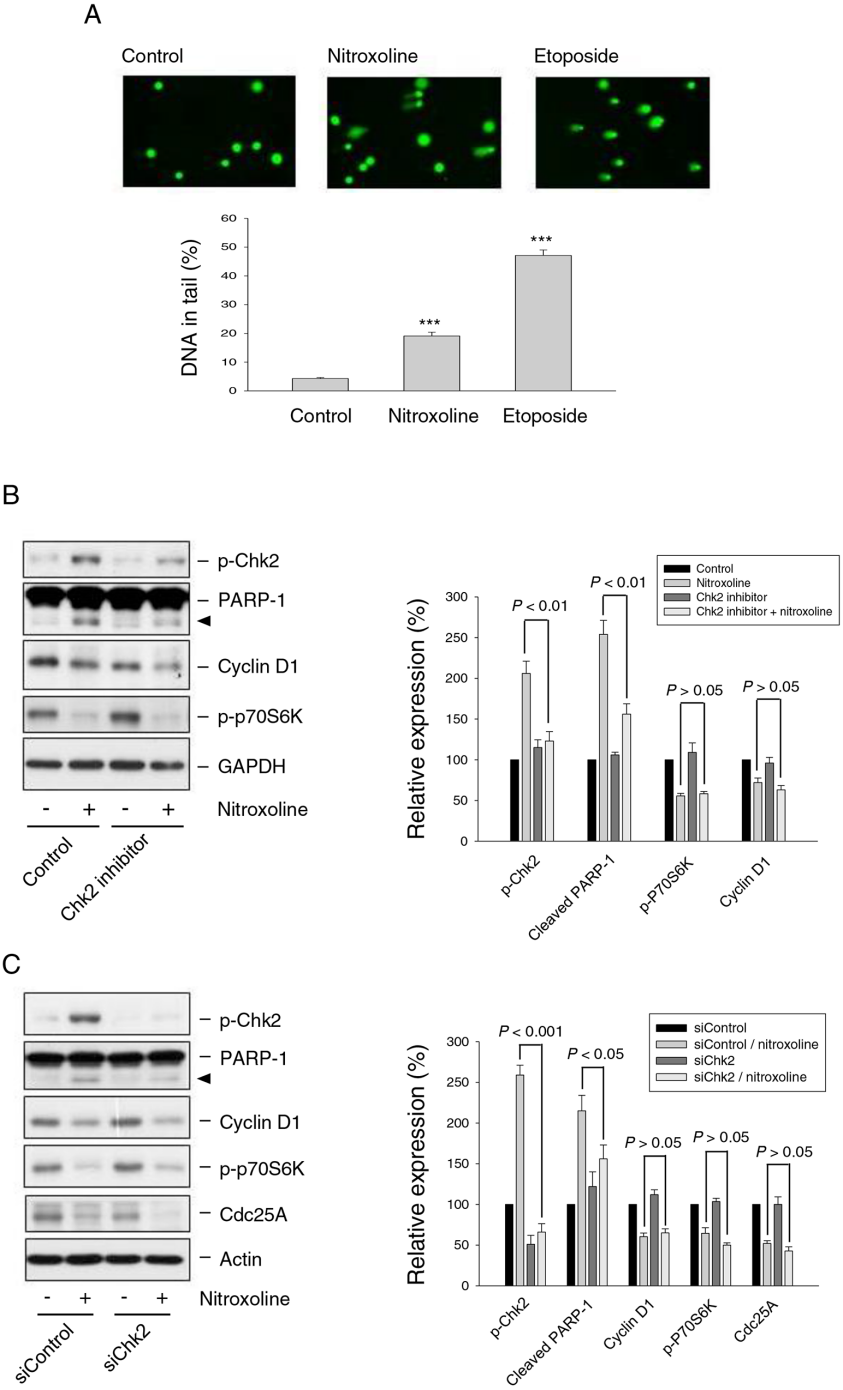
Effect of nitroxoline on DNA damage response and examination of Chk2-mediated protein expression **A.** PC-3 cells were incubated in the absence or presence of nitroxoline (10 μM) or etoposide (50 μM) for 12 hours. Comet assay was employed to examine the integrity of chromosome DNA. Comet tail-positive cells indicate under DNA damage stress. Data are expressed as mean ± SEM of three independent determinations. ***P < 0.001 compared with the control. **B and C.** PC-3 cells were pre-treated with or without Chk2 inhibitor (20 μM) or transfected with siChk2. The cells were incubated in the absence or presence of nitroxoline (10 μM) for 24 hours. Cells were harvested and lysed for the detection of the protein expression using Western blot analysis. The protein expression was quantified using computerized image analysis system ImageQuant (Amersham Biosciences, NJ, USA). Data are expressed as mean ± SEM of three independent experiments.

Chk2 and Chk1 pathways that are activated by DNA double-strand breaks and single-stranded DNA, respectively, coordinate cellular responses to DNA damage [29]. Nitroxoline induced the phosphorylation and activation of Chk2 other than Chk1 in both LNCaP (Supplementary Figure 1) and PC-3 cells (Figure 3A and 3B), which were AMPK-dependent since the effect was significantly reduced by AMPKα knockdown (Figure 4). Recent work has revealed DNA damage-independent function of Chk2 involving in mitotic spindle assembly and chromosomal stability [30]. The data showed that nitroxoline resulted in a significant increase of DNA damage using comet assay (Figure 5A). The data suggest DNA damage-dependent function of Chk2. Activated Chk2 kinase may serve as a signal transducer in promoting cell survival or death. It phosphorylates numerous substrates including p53, E2F, Cdc25 phosphatases and BRCA1 which are related to halting cell cycle progression, introducing DNA repair, and inducing apoptosis following DNA damage response [30]. The application of both pharmacological Chk2 inhibitor and siRNA to knockdown Chk2 did not prevent nitroxoline-induced cyclin D1 down-regulation, p70S6K inhibition and Cdc25A down-regulation, but significantly blunted the cleavage of PARP-1 (Figure 5B and 5C), supporting a pro-apoptotic role of Chk2. The data were confirmed using SRB assay.

**Figure 6:**
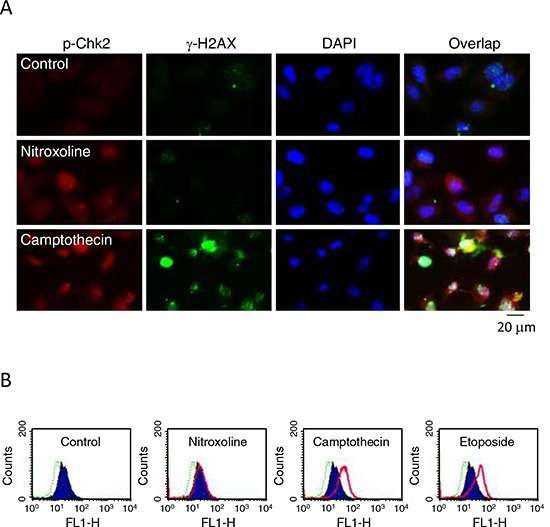
Effect of nitroxoline on protein expression of Chk2 and γ-H2AX. PC-3 cells were incubated in the absence or presence of nitroxoline (10 μM), camptothecin (10 μM) or etoposide (25 μM) for 24 hours **A.** The cells were fixed for immunofluorescence microscopic examination by staining with anti-Chk2 (red fluorescence) and anti-γ-H2AX (green fluorescence) antibodies and DAPI (blue fluorescence, for nuclear detection). *Scale bar*, 20 μm. **B.** The cells were harvested for detection of γ-H2AX using flow cytometric analysis. Dashed line area, basal; blue area, vehicle control; pink area, the indicated drug. The data are representative of two independent experiments.

H2AX, a variant of the H2A protein family, is phosphorylated by Ataxia telangiectasia mutated (ATM)- and Rad3-related protein (ATR) when DNA damage forms double-strand breaks. The newly phosphorylated H2AX, γ-H2AX, is the initial step in recruiting DNA repair proteins [29, 31, 32]. Both immunofluorescence microscopic examination and flow cytometric analysis showed that nitroxoline did not induce γ-H2AX formation but increased Chk2 phosphorylation. In contrast, camptothecin and etoposide which were DNA damage inducers through inhibition of topoisomerase I and II, respectively, caused increased levels of γ-H2AX and Chk2 phosphorylation (Figure 6A and 6B). The data suggest the absence of γ-H2AX-related DNA repair mechanism to nitroxoline action.

### ZnCl_2_ supplement does not rescue nitroxoline-mediated anti-proliferative effect and cellular signaling

**Figure 7:**
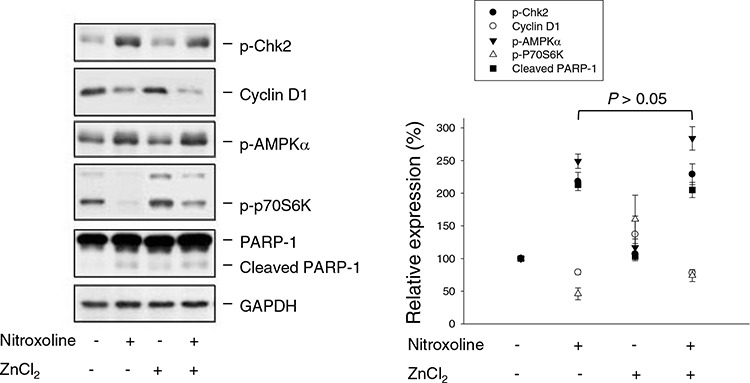
Effect of ZnCl_2_ supplementation on nitroxoline-induced effect PC-3 cells were incubated in the absence or presence of ZnCl_2_ (100 μM) or nitroxoline (10 μM) for 24 hours. Cells were harvested and lysed for the detection of protein expression using Western blot analysis. Expression levels were quantified using computerized image analysis system ImageQuant (Amersham Biosciences, NJ, USA). Data are expressed as mean ± SEM of three independent experiments.

Nitroxoline is an antibiotic functioning by chelating Fe^2+^ and Zn^2+^. Zn^2+^ serves as structural ions in transcription factors and is a cofactor in more than 300 enzymes affecting a wide variety of cellular functions [33]. The re-introduction of Zn^2+^ was applied to determine whether the ions chelating effect contributed to nitroxoline-induced anticancer activity. As a result, ZnCl_2_ supplement did not significantly prevent Chk2 activation, cyclin D1 down-regulation, AMPK activation, p70S6K inhibition and PARP-1 cleavage (Figure 7). SRB assay also showed that ZnCl_2_ supplement did not modify nitroxoline-induced anti-proliferative effect (IC_50_ of 6.1 ± 0.6 μM *vs*. 5.5 ± 0.1 μM of nitroxoline alone, *n* = 4, *P* > 0.05).

## DISCUSSION

Nitroxoline, an FDA-approved antibiotic, has been identified with potential anticancer activity in both *in vitro* and *in vivo* studies [17–19]. In this study, nitroxoline induced anti-proliferative effects in both hormone-sensitive and hormone-refractory prostate cancers through induction of G1 arrest of the cell cycle. Cyclin D1 is a specific G1 phase protein forming a complex with and serving as a regulatory subunit of Cdk4 or Cdk6. Overexpression of cyclin D1 has been detected in numerous types of cancers, including cancers of the breast, lung and prostate [34–36] and has been considered as an oncogenic mechanism in hormone-refractory metastatic prostate cancer to the bone [36]. Rb is a critical tumor suppressor for G1 checkpoint, inhibiting the entry of cell cycle into S phase. Cyclin D1/Cdk4 complex can partially and appropriately phosphorylate Rb protein, leading to its dissociation from E2F1 transcription factor and allowing the activation of gene transcription [20, 21]. Many cancers have been shown to exhibit chromosomal abnormalities which directly result in the functional inactivation of Rb and promote cell proliferation [37]. In the present work, nitroxoline induced a dramatic down-regulation of cyclin D1 expression and inhibition of Rb phosphorylation that explained the G1 arrest of cell cycle and indicated the potential against prostate cancers.

mTOR, a serine/threonine kinase, plays a crucial role in cell proliferation, growth, survival and metabolism through the regulation of multiple interacting proteins. mTORC1 is a major rapamycin-sensitive mTOR complex, promoting protein synthesis in response to growth factors through the phosphorylation of p70S6K and 4EBP1 [25, 38]. p70S6K activation involves the phosphorylation of several serine and threonine sites; the critical event is Thr^389^ phosphorylation. Both phosphoinositide 3-dependent protein kinase 1 and mTOR are potent Thr^389^ kinases [39, 40]. Pharmacogenomic profiling of mTOR-p70S6K pathway shows that the protein synthetic machinery is overexpressed and activated during tumor progression [41]. Immunohistochemistry analysis also reveals a strong association between mTOR-p70S6K activation and prostate cancer progression [42, 43]. During G1 phase, cells grow in size and synthesize mRNA and proteins required for DNA synthesis at S phase. Nitroxoline displayed profound inhibition of mTOR-p70S6K pathway supporting G1 arrest activity. AMPK, a cellular energy sensor and signal transducer, is a key player to switch off mTOR pathway through phosphorylating and thereby activating tuberous sclerosis protein (TSC) 2, leading to association of TSC1/TSC2 complex and inhibition of mTOR pathway. AMPK is a potential target of drugs for regulating numerous human diseases ranging from metabolic disorders to cancers [25, 44]. AMPK knockdown significantly prevented the inhibition of mTOR-p70S6K pathway and rescued the cyclin D1 expression, indicating the central role of AMPK in nitroxoline-mediated effect.

Nitroxoline also induced autophagy through an AMPK-dependent pathway. Recent studies suggest that Atg1/UNC-51-like autophagy activating kinase 1 (Ulk 1) complex is an essential player in autophagy initiation [45]. Ulk1, a homologue of yeast Atg1, is activated by AMPK via direct phosphorylation of Ser^317^ and Ser^777^. In contrast, mTOR phosphorylates Ulk1 at Ser^757^ and interrupts the interaction between AMPK and Ulk1 [46]. Coordination between AMPK and mTOR is critical for Ulk1 in autophagy induction and may explain nitroxoline-induced activation of AMPK and autophagy. However, the increased autophagy decreased apoptosis to nitroxoline action. The cytoprotective autophagy has been studied under cellular stresses, such as hypoxia, nutrient deprivation, oxidative stress and cytotoxic drug exposure. Therefore, autophagy advances survival and growth of cancer cells and resistance to chemotherapy [27, 47]. Targeting autophagy in cancer will provide novel strategy for drug development [47, 48].

Chk2 encodes for a serine/threonine kinase which coordinates cellular responses to DNA damage [29]. It phosphorylates diverse substrates, including p53, E2F and Cdc25 phosphatases, associated with the arrest of cell cycle and introduction of DNA repair [3, 30]. More specifically, Chk2 is able to phosphorylate and down-regulate Cdc25A, resulting in G1 arrest of the cell cycle [49]. However, the data showed that Chk2 activation contributed to nitroxoline-induced cleavage of PARP-1 (a caspase-3 substrate) other than the regulation of cyclin D1, Cdc25A and p70S6K. The data indicate that Chk2 serves as a pro-apoptotic activator but not a coordinator in cell cycle arrest. Recent work suggests that γ-H2AX plays a key role in the enrollment and maintenance of DNA repair and checkpoint proteins [29, 31, 32]. It has been reported that H2AX^−/−^ mouse embryonic fibroblasts are susceptible to radiation, showing deficits in repairing DNA damage compared to the wild-type counterparts [32]. The present study showed that nitroxoline, at effective concentrations in displaying anti-proliferative and apoptotic activities, did not induce γ-H2AX formation and suggested the absence of γ-H2AX-related DNA repair mechanism. However, higher concentrations of nitroxoline could induce γ-H2AX formation (data not shown) and, therefore, the related DNA repair mechanism might be activated. Notably, the activation of Chk2 was AMPK-dependent in prostate cancers upon nitroxoline exposure. Recent work with AMPKα knockout mouse embryonic fibroblasts has demonstrated a reduced phosphorylation and activity of Chk2 kinase in response to ionizing radiation [50]. Moreover, AMPK has been suggested to function in a point of convergence of metabolic and genomic stress signals, regulating downstream signaling upon growth factor stimulation and propagating DNA damage response through ATM/Chk2 pathway [51]. These studies support an upstream role of AMPK in regulating Chk2 activity. However, defect in DNA repair mechanism during nitroxoline treatment shifted the pro-survival nature of Chk2 into pro-apoptotic disposition. Similar pro-apoptotic function of Chk2 also has been suggested. Small molecules like Nutlin-3 and RITA (reactivation of p53 and induction of tumor cell apoptosis) have been reported to induce apoptosis in human uveal melanoma cells and colorectal carcinomas through p53-dependent Chk2 activation [52]. In contrast, Chk2 expression in p53-mutated cancer cells, in spite of the absence of DNA damaging stimuli, is sufficient to induce apoptosis [53]. These studies suggest that direct activation of Chk2 can be explored as a novel anticancer approach.

**Figure 8:**
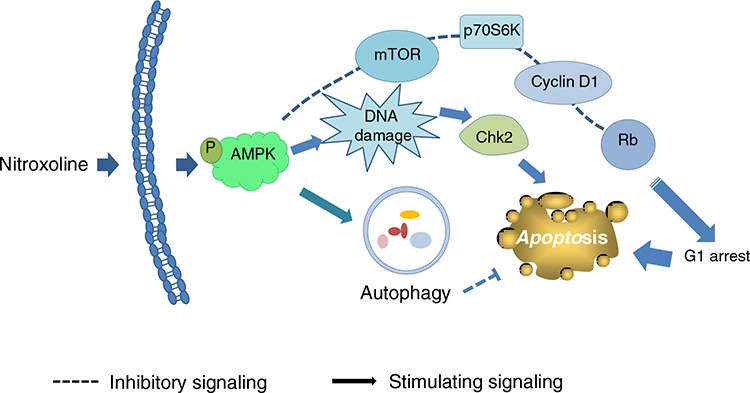
Schematic figure for nitroxoline-mediated signaling pathways on the inhibition of cell proliferation and apoptosis Nitroxoline induces the activation of AMPK, which *in turn* inhibits mTOR signaling pathway and cyclin D1-Rb-Cdc25A axis, leading to G1 arrest of cell cycle and apoptosis. Nitroxoline also induces AMPK-dependent activation of Chk2 contributing to cell apoptosis. However, the activated autophagy may blunt the apoptosis.

Taken together, the data suggest nitroxoline induces anti-proliferative and apoptotic signaling cascades against prostate cancer in a sequential manner (Figure 8). Nitroxoline induces the activation of AMPK, which *in turn* inhibits mTOR signaling pathway and cyclin D1-Rb-Cdc25A axis, leading to G1 arrest of cell cycle and apoptosis. Nitroxoline also induces AMPK-dependent activation of Chk2 that, at least partly, contributes to cell apoptosis. However, the activated autophagy may blunt apoptosis. The data suggest the potential of nitroxoline for therapeutic development against prostate cancer. Inhibitors of autophagy may potentiate apoptotic cell death and provide novel strategy for the development of nitroxoline.

## MATERIALS AND METHODS

### Materials

RPMI 1640 medium and fetal bovine serum (FBS) were obtained from GIBCO/BRL Life Technologies (Grand Island, NY). Antibodies to cyclin D1, cyclin E, cyclin A, cyclin B1, Cdk4, Cdk2, Cdk1, p-Rb^Ser807/811^, Cdc25A, PARP-1, AMPK siRNA and anti-mouse and anti-rabbit IgGs were obtained from Santa Cruz Biotechnology, Inc. (Santa Cruz, CA). Antibodies to p-Chk1^Ser345^, p-Chk2^Thr68^, p-AMPKα^Thr172^, p-mTOR^Ser2448^, p-p70S6K^Thr389^ (p70 ribosomal S6 kinase), p62, p-H2AX^Ser139^ and GAPDH were from Cell Signaling Technologies (Boston, MA). Antibody against LC3 was purchased from Novus Biologicals (Littleton, CO). The siRNA specifically targeting Chk2 mRNA was purchased from Dharmacon (Lafayette, CO). SRB, PI, CFSE, 4,6-diamidino-2-phenylindole dihydrochloride (DAPI), nitroxoline, paclitaxel, etoposide, Chk2 inhibitor and all other chemical compounds were obtained from Sigma-Aldrich (St. Louis, MO).

### Cell lines and cell culture

PC-3, DU-145 (hormone-refractory) and LNCaP (hormone-sensitive) prostate cancer cell lines were from American Type Culture Collection (Rockville, MD). Cells were cultured in RPMI 1640 medium with 10% FBS (*v/v*), penicillin (100 units/ml) and streptomycin (100 μg/ml). Cultures were maintained in a 37°C incubator with 5% CO_2_.

### SRB assay

Cells were seeded in 96-well plates in medium with 10% FBS. After 24 hours, cells were fixed with 10% trichloroacetic acid (TCA) to represent cell population at the time of agent addition (T_0_). After additional incubation of 0.1% dimethylsulfoxide (DMSO) or the agent for 48 hours, cells were fixed with 10% TCA and SRB at 0.4% (w/v) in 1% acetic acid was added to stain cells. Unbound SRB was washed out by 1% acetic acid. SRB bound cells were solubilized with 10 mM Trizma base. The absorbance was read at a wavelength of 515 nm. Using the following absorbance measurements, such as time zero (T_0_), control growth (C), and cell growth in the presence of the agent (Tx), the percentage growth was calculated at each of the agent concentrations levels. Percentage growth inhibition was calculated as: [1-(Tx-T_0_)/(C-T_0_)] × 100%. Growth inhibition of 50% (IC_50_) is determined at the agent concentration which results in 50% reduction of total protein increase in control cells during the agent incubation.

### Cell proliferation assay with CFSE labeling

The cells were treated with CFSE at a final concentration of 10 μM. After incubation at 37°C for 10 minutes, RPMI medium with 10% FBS was added. Tubes were placed in ice for 5 minutes and then washed. After centrifugation, the cells were seeded in RPMI medium with 10% FBS for 24, 48 and 72 hours at 37°C under 5% CO_2_/95% air. After treatment, the fluorescence intensity was determined by flow cytometric analysis (Becton Dickinson, Mountain View, CA).

### Colony formation assay

To assess anchorage-dependent colony formation effect, the cells (100 cells/well) were seeded in a 6-well plate. After a 10-day treatment with nitroxoline, the cell colonies were rinsed with phosphate-buffered saline (PBS), stained with 0.25% crystal violet/20% ethanol and photographed by a camera with Copy Stan (Nikon Model No. CS-920; Shimadzu, Japan).

### Flow cytometric analysis of PI staining

Cells were harvested by trypsinization, fixed with 70% (v/v) alcohol at 4°C for 30 minutes and washed with PBS. After centrifugation, cells were incubated in 0.1 M phosphate-citric acid buffer (0.2 M NaHPO_4_, 0.1 M citric acid, pH7.8) for 30 minutes. Cells were centrifuged and resuspended with 0.5 ml PI solution containing Triton X-100 (0.1% v/v), RNase (100 μg/ml) and PI (80 μg/ml). DNA content was analyzed with FACScan and CellQuest software (Becton Dickinson, Mountain View, CA).

### DNA fragmentation assay

The DNA fragmentation was determined using the Cell Death Detection ELISAplus kit (Roche, Mannheim, Germany). The assay was based on the quantitative *in vitro* determination of cytoplasmic histone-associated DNA fragments (mono- and oligonucleosomes) after induced cell death. After the treatment, the cells were lysed and centrifuged, and the supernatant was used for the detection of nucleosomal DNA according to the manufacturer's protocol.

### Western blotting

After treatment, the cells were washed twice with ice-cold PBS and reaction was terminated by the addition of 100 μl ice-cold lysis buffer (10 mM Tris-HCl, pH 7.4, 150 mM NaCl, 1 mM EGTA, 1 mM PMSF, 10 μg/ml aprotinin, 10 μg/ml leupeptin, and 1% Triton X-100). For western blot analysis, the amount of proteins (40 μg) were separated by electrophoresis in a 10% polyacrylamide gel and transferred to a nitrocellulose membrane. After an overnight incubation at 4°C in PBS/5% nonfat milk, the membrane was washed with PBS/0.1% Tween 20 for 1 hour and immuno-reacted with the indicated antibody for 2 hours at room temperature. After four washings with PBS/0.1% Tween 20, the anti-mouse or anti-rabbit IgG (dilute 1:2000) was applied to the membranes for 1 hour at room temperature. The membranes were washed with PBS/0.1% Tween 20 for 1 hour and the detection of signal was performed with an enhanced chemiluminescence detection kit (Amersham Biosciences, NJ, USA).

### Small interfering RNA (siRNA) transfection

Cells were seeded into 60-mm tissue culture dishes with 30% confluence and grown for 24 hours to 50% confluence. Each dish was washed with serum-free Opti-MEM (Life Technologies, Ground Island, NY), and 2 ml of same medium was added. Aliquots containing siRNA in serum-free Opti-MEM were transfected into cells using Lipofectamine 2000 according to the instructions. After incubation for 24 to 48 hours, cells were washed with medium and incubated in 10% FBS-containing RPMI-1640 medium for 24 hours. The cells were treated with or without nitroxoline and the protein expression was detected using Western bot analysis.

### Comet assay

After treatment, the cells were pelleted and resuspended in ice-cold PBS. The resuspended cells were mixed with 1.5% low melting point agarose. This mixture was loaded onto a fully frosted slide that had been pre-coated with 0.7% agarose and a coverslip was then applied to the slide. The slides were submerged in pre-chilled lysis solution (1% Triton X-100, 2.5 M NaCl, and 10 mM EDTA, pH 10.5) for 1 hour at 4°C. After soaking with pre-chilled unwinding and electrophoresis buffer (0.3 N NaOH and 1 mM EDTA) for 20 minutes, the slides were subjected to electrophoresis for 15 minutes at 0.5 V/cm (20 mA). After electrophoresis, slides were stained with 1X Sybr Gold (Molecular Probes) and nuclei images were visualized and captured at 400X magnifications with an Axioplan 2 fluorescence microscope (Zeiss, Germany) equipped with a CCD camera (Optronics, Goleta, CA). Over hundreds of cells were scored to calculate the overall percentage of comet tail-positive cells.

### Immunofluorescence microscopic examination

After treatment, the cells were fixed with 100% methanol at −20°C for 20 minutes and incubated in 1% bovine serum albumin containing 0.1% Triton X-100 at 37°C for 30 minutes. For double staining, cells were first processed the single staining procedure. The cells were washed twice with PBS for 5 minutes and stained with the p-chk2 antibody at room temperature for 1 hour and then, the Cyt3-conjugated secondary antibody at room temperature for 1 hour. After washed with PBS, this process was repeated for staining γ-H2AX with FITC-conjugated secondary antibody. Nuclear staining was performed by 1 μg/ml DAPI. The cells were analyzed by an Axio Imager A1 microscope (Carl Zeiss).

### Flow cytometric assay of γ-H2AX

After treatment, the cells were harvested by trypsinization, fixed with 70% (v/v) ethanol at −20°C for 30 minutes and washed with PBS. After centrifugation, cells were permeabilized with 0.1% Triton X-100/PBS and then incubated with anti-γ-H2AX antibody for 1 hour at room temperature. The cells were washed and incubated with FITC-labeled anti-rabbit secondary antibody for 1 hour at room temperature. Cells were washed and re-suspended in PBS for the analysis with FACScan and CellQuest software (Becton Dickinson, Mountain View, CA).

### Data analysis

Data are presented as the mean ± SEM for the indicated number of independent experiment. Statistical analysis of data for multiple groups is performed with one-way analysis of variance (ANOVA). Student's *t*-test is applied for comparison of two groups. *P*-values less than 0.05 are statistically considered significant.

## SUPPLEMENTARY FIGURES


