# Supplementary material for: Repurposing of nitroxoline as a potential anticancer agent against human prostate cancer – a crucial role on AMPK/mTOR signaling pathway and the interplay with Chk2 activation
Source: Oncotarget. 2015 Oct 3;6(37):39806–20. doi: 10.18632/oncotarget.5655 (PMC4741862; doi:10.18632/oncotarget.5655)
Supplement: Supplementary file 1 [file oncotarget-06-39806-s001.pdf]

## SUPPLEMENTARY FIGURES

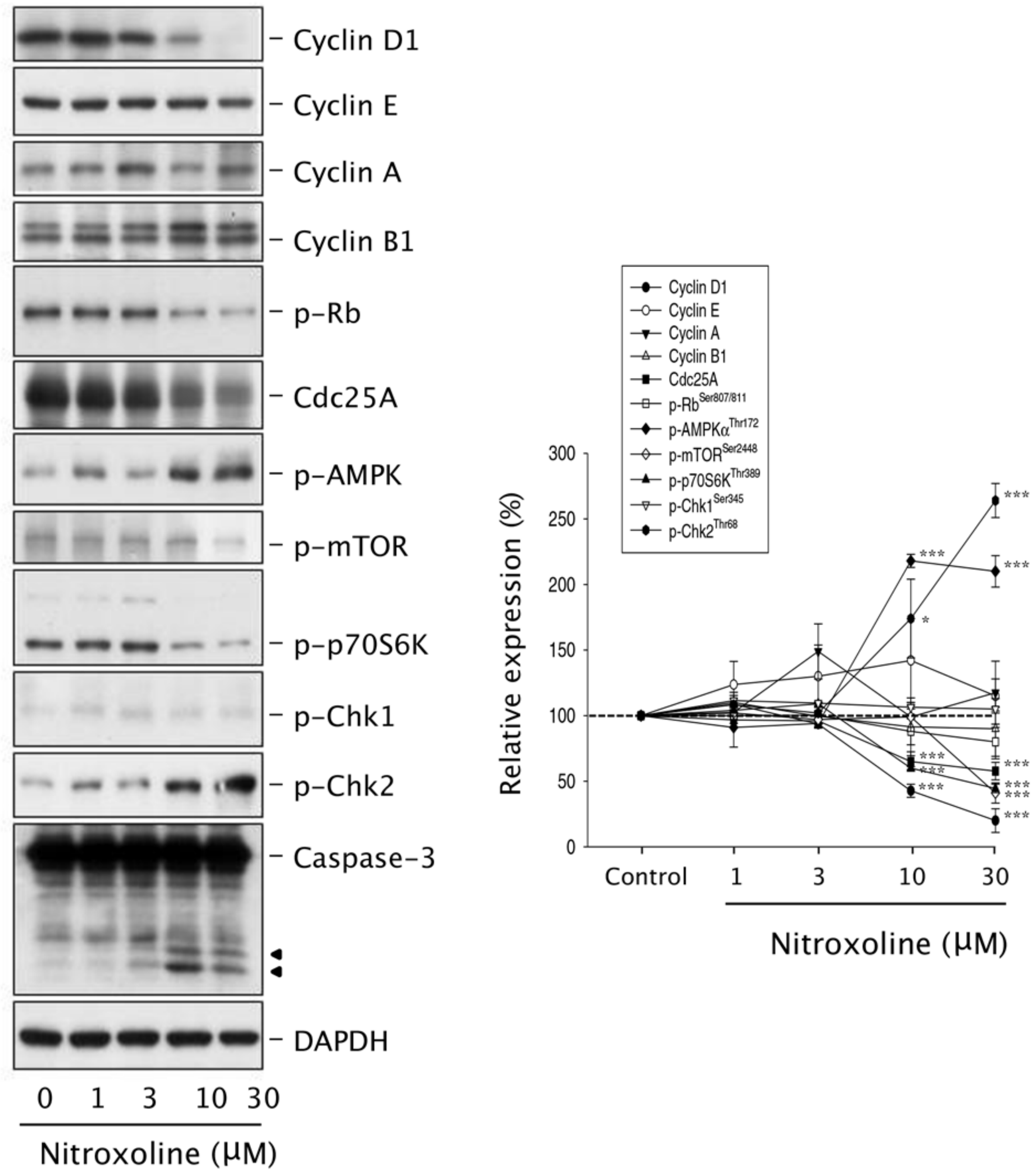


**Supplementary Figure S1: Effect of nitroxoline on the expression of several proteins.** LNCaP cells were incubated in the absence or presence of nitroxoline (1 to 30 μM) for 24 hours. Cells were harvested and lysed for the detection of the indicated protein expression by Western blot analysis. Protein expression was quantified using computerized image analysis system ImageQuant (Amersham Biosciences). Data are expressed as mean ± SEM of three independent experiments. *$P < 0.05$ and ***$P < 0.001$ compared with the control.

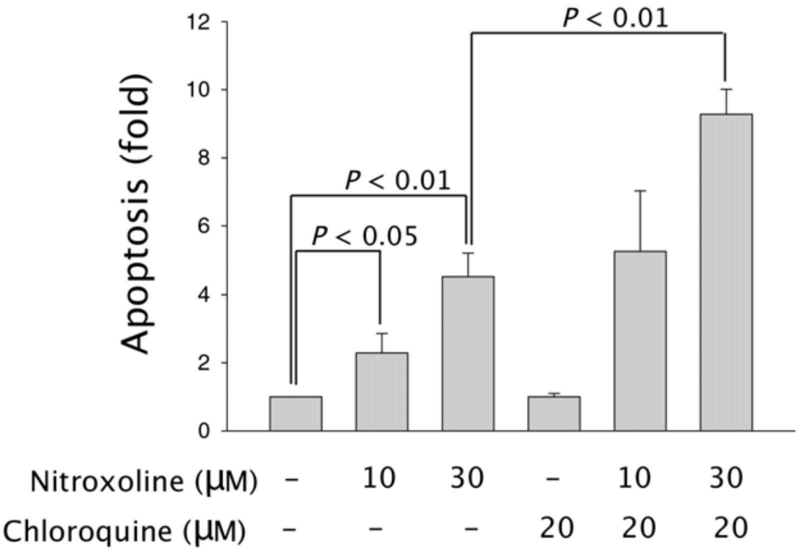


**Supplementary Figure S2: Combinatory effect of nitroxoline and chloroquine on cell apoptosis.** PC-3 cells were incubated in the absence or presence of the indicated compound for 48 hours. The cells were harvested for the determination of apoptosis based on the quantitative *in vitro* determination of cytoplasmic histone-associated DNA fragments (mono- and oligonucleosomes). Data are expressed as mean ± SEM of three independent experiments.
